# Adherence to published guidelines for perioperative care of the elderly: a survey of Scottish anaesthetic departments

**DOI:** 10.1186/s13741-022-00258-z

**Published:** 2022-07-05

**Authors:** Clair Clark, Ella Bennett, Irwin Foo

**Affiliations:** Anaesthetics Department, Western General Hospital, Crewe Road South, Edinburgh, EH4 2XU UK

**Keywords:** Perioperative, Frailty, Geriatric, Preoperative assessment, Cognitive impairment, Delirium

## Abstract

**Background:**

In 2010, a national enquiry into elderly patient outcomes after surgery identified that only 36% received ‘good’ care. Guidance was subsequently published by the Association of Anaesthetists of Great Britain and Ireland regarding perioperative care of the elderly and those with dementia; this study aims to assess current adherence to these guidelines in anaesthetic departments across Scotland.

**Methods:**

A web-based survey was sent to all Scottish departments. The questions assessed department patient demographic, access to specialist pre-assessment services, availability of multidisciplinary input, perioperative care of patients with cognitive impairment and departmental training on geriatric perioperative care.

**Results:**

Responses were collected from November-December 2020 with a 92.6% response rate. A total of 64% of departments stated that > 50% of their workload involved patients over 75. One department had a lead clinician for geriatric anaesthesia, whilst 20% could access a geriatric specialist when coordinating perioperative care. Specialist geriatric pre-assessment services operate in 20% of centres. A total of 60% of respondents used a clinical frailty score when pre-assessing patients over 75, with 48% specifically screening for cognitive impairment. The vast majority of centres, 76%, did not routinely provide information regarding post-operative delirium and 24% ‘never or very rarely’ invite caregivers to accompany patients with dementia into the department. Education sessions regarding perioperative elderly care had occurred in 56% of departments.

**Conclusions:**

Elderly patients represent a significant proportion of anaesthetic workload in Scotland. Despite this, adherence to recommended practice is low. The vast majority of centres lack access to specialist multidisciplinary input or specialist pre-assessment services which are essential to providing good care. Reported screening for frailty and cognitive impairment is variable, with opportunities for improvement in communication and education (patient and clinician) surrounding these conditions.

**Supplementary Information:**

The online version contains supplementary material available at 10.1186/s13741-022-00258-z.

## Background

It is well established, across the developed world, that our population is ageing with the over 65 cohort making up an increasing percentage of the population as a whole. As mortality and fertility rates continue to drop, it is predicted that by 2050, 1 in 4 people in the UK will be aged over 65 (Office for National Statistics 2021). Consequently, an increasing proportion of anaesthetic workload is going to be in patients from within this cohort. In 2010, the National Confidential Enquiry into Patient Outcome and Death (NCEPOD) ‘Elective and Emergency Surgery in the Elderly: An Age Old Problem’ identified that despite the publication of a framework of care for geriatric patients over a decade earlier, there had been little improvement in patient care. Indeed, only 36% of patients over 80 years old received care that the advisors could describe as ‘good’ (National Confidential Enquiry into Patient Outcome and Death 2010).

Morbidity after surgery in the older patient include not only direct organ dysfunction but also much higher rates of post-operative delirium, cognitive decline and progression of frailty. Multidisciplinary care and protocolised pathways improve these outcomes (Association of Anaesthetists of Great Britain and Ireland 2014). The Association of Anaesthetists of Great Britain and Ireland (AAGBI) published a safety guideline in 2014 for the perioperative care of the elderly containing a number of recommendations aimed at improving patient care and reducing morbidity and mortality (Association of Anaesthetists of Great Britain and Ireland 2014). The AAGBI guideline also recognised that elderly patients with dementia and cognitive decline are particularly vulnerable because of issues surrounding communication and delayed presentation as well as the direct effects of anaesthesia on cognitive function. Therefore, a guideline for the perioperative care of patients with dementia was published in 2019 with the aim of improving care in this complex cohort (White et al. 2019). Whether both these guidelines are being implemented in current practice, however, remains unclear. A recent study of American Anaesthesiologists identified that despite the availability of guidelines, practice varied greatly (Deiner et al. 2020). The aim of this survey was to assess whether perioperative care of patients over the age of 75 in Scotland adhered to AAGBI guidance.

## Methods

### Questionnaire construction

We constructed ten questions, using the two guidelines as a framework which covered the key AAGBI recommendations promoting optimum perioperative care of elderly patients. These questions pertain mostly to the preoperative period. Our first question establishes the proportion of each department’s annual caseload that involves patients aged over 75. The following three questions address whether departments have preoperative services and multidisciplinary input specifically for patients aged over 75. Our next three questions ask specifically about screening for cognitive impairment and frailty preoperatively and whether this is communicated to patients and their carers. The remaining questions address the care of patients with dementia in the immediate perioperative period and whether departments have conducted any training on the perioperative care of older patients undergoing anaesthesia and surgery.

To collect responses, we used a combination of binary answers with the addition of an ‘unsure’ option and a 5-point Likert scale with the following options: nearly all of the time (greater than 95%), usually (about 75%), sometimes (about 50%), occasionally (about 25%) and never or very rarely (less than 5%). These questions were then compiled into an online survey via the SurveyMonkey web platform.

### Data collection and analysis

In order to distribute the survey across Scotland, we contacted each hospital with a theatre department, via the anaesthetic secretary, to ascertain the most appropriate individual to contact. This was an anaesthetist that either had formal responsibility or a special interest in the perioperative care of elderly patients. Where no specific anaesthetist fulfilled these criteria, we asked for an anaesthetist involved in preoperative assessment services or a general interest in elderly patient anaesthesia. Each anaesthetist was then sent an email explaining the purpose of the survey and a link to access it online. Follow-up emails were sent to initial nonrespondents over a 3-week period with paper copies then posted to those who still had not responded. No incentives were provided for participation in the survey. Data was analysed using descriptive statistics and Mann-Whitney *U*-tests where appropriate.

In a number of instances, the respondent had chosen the option ‘unsure’ but then using free text comments explained their specific departmental situation. Often, this allowed for clarification such that their answer could actually be changed to ‘yes’ or ‘no’. As an example, responses which were marked as ‘unsure’ were accompanied by free text explanation of ‘not that I am aware of’ or ‘in discussion’. In such circumstances, these answers were changed to ‘no’.

## Results

Departments across Scotland were first contacted in October 2020, with follow-up emails and paper questionnaires sent by November 2020. The survey was closed in December 2020, when 25 of the 27 centres had submitted a response (92.6%). Of the two centres that did not complete the survey, one did not respond, and the other replied stating that they were unable to participate due to increased departmental workload during the COVID-19 pandemic. Departments were subsequently grouped according to the number of inpatient beds they have — group 1 had fewer than 150 beds, group 2 between 150 and 400 beds, group 3, 400 to 700 beds and group 4 hospital had greater than 700 inpatient beds. Group 1 are classified by the Scottish government as rural general hospitals (The Scottish Government 2008).

**Fig. 1:**
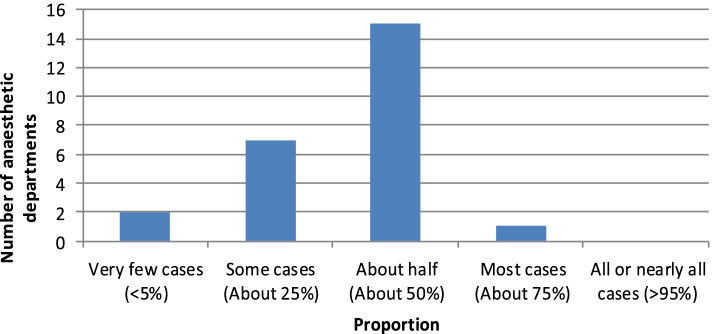
Proportion of departmental caseload involving patients aged 75 across Scotland

Of the respondents, 64% confirmed that a majority (50–75%) of their workload consisted of patients over the age of 75 (Fig. 1). Despite this, only one department stated that they had a lead clinician for perioperative care of the elderly. Furthermore, only 20% of centres had access to a geriatric specialist when coordinating perioperative care of this group of patients. This trend continued with pre-assessment services for the older patient, which was available only in 20% of centres. Unsurprisingly, the centre with a lead clinician for geriatric anaesthesia had both a specialist pre-assessment service and access to a geriatrician for advice on perioperative care. A larger department did not infer better services; only one of the group 4 hospitals (greater than 700 inpatient beds) offered this service.

**Fig. 2:**
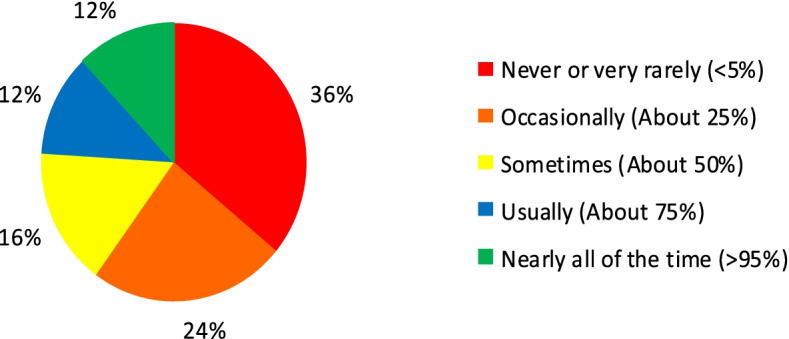
Frequency that the risk of post-operative delirium and cognitive decline is discussed with patients and their carers

Adherence to guidance regarding the perioperative care of frail patients and those with cognitive impairment was variable. A total of 60% of respondents stated that they used a recognised clinical frailty score (CFS) when pre-assessing older adults. Additionally, 48% reported that they specifically screen adults over 75 years for cognitive impairment. However, the vast majority (76%) of centres did not routinely provide patients and/or carers with information regarding the risk of post-operative delirium (Fig. 2). When treating patients with moderate to severe dementia, 44% reported that carers or family members were invited to accompany the patient ‘frequently’ (75% of the time) or ‘nearly always’; however, 24% of participants stated that this happened ‘never or very rarely’ (less than 5% of the time).

Hospital size did appear to influence whether cognitive screening occurred in older patients; only 2 of the groups 1 and 2 hospitals (18%) frequently screen for cognitive impairment, compared with 10 of the groups 3 and 4 hospitals (71%).

To allow for further comparison based on hospital size, we translated the responses regarding communication of the risk of post-operative delirium (or worsening of existing impairment) into a linear scale as follows: never or very rarely = 1, occasionally = 2, sometimes = 3, usually = 5 and nearly all of the time = 5. This resulted in an average response of ‘2’ for groups 1 and 2 hospitals compared with ‘2.7’ for groups 3 and 4 hospitals. This difference however, was not statistically significant (*p=*0.312). When the responses regarding inviting carers or family to accompany vulnerable patients into a department were similarly converted, groups 1 and 2 hospitals scored an average of 2.09, whilst groups 3 and 4 hospitals scored an average of 3.64 (*p* = 0.010).

Education sessions on the perioperative care of the older patient occurred in 56% of departments. This question was framed to include the last 2 years as it was felt that teaching opportunities last year may have been limited by the COVID-19 pandemic.

## Discussion

Among the 25 (out of 27) Scottish centres that responded to the survey, 64% stated that the majority of their workload involved the perioperative care of older patients. This is consistent with the findings of a similar survey by the American Society of Anesthesiologists (ASA), where 60% of their members reported that at least half of their cases were elderly adults (Deiner et al. 2020). This is unsurprising as our population is ageing, and it is well-established that although adults over 75 accounted for less than 10% of the population, they represented nearly 25% of all operations performed (Association of Anaesthetists of Great Britain and Ireland 2014). Despite this, and the AAGBI’s 2014 recommendation, only one department had an appointed lead clinician for geriatric anaesthesia. Furthermore, this centre with the lead clinician had both a specialist pre-assessment service and access to a geriatrician for advice on perioperative medicine.

There is a large body of evidence which suggests that multidisciplinary care improves both perioperative morbidity and mortality in elderly patients (Association of Anaesthetists of Great Britain and Ireland 2014). Paramount to this is the inclusion of a senior geriatrician in the co-ordination of perioperative care, a recommendation that is echoed in guidelines across specialities, from the Royal College of Emergency Medicine to the British Geriatrics Society (BGS) (Conroy 2012; Dhesi 2018). Only 20% of departments had access to this, a finding that highlights a fundamental barrier to delivering good multidisciplinary care. It must be acknowledged, however, that the design of our survey relied on a nominated individual replying on behalf of their department. In many cases, this individual had no formal responsibility for geriatric anaesthesia, and a few had no special interest in geriatrics but declared interest in pre-assessment. This leaves the data vulnerable to individual knowledge (or lack thereof) with potential to miss less formal multidisciplinary team (MDT) relationships that would lead to under-reporting of the available specialist input. In addition to this, the survey will not identify any formal collaboration between surgeons and geriatricians, a model that is increasingly seen in areas such as ortho-geriatrics and enhanced recovery after surgery (ERAS) programmes. It is, however, still useful in indicating that the formal clinical collaboration between anaesthetics and geriatricians, recommended by both the AAGBI and BGS, is clearly not yet established and still in its infancy (Association of Anaesthetists of Great Britain and Ireland 2014; Conroy 2012).

It is difficult to explain why there appear to be so few formal anaesthetic-geriatric care pathways, and this may simply reflect more informal surgical-geriatric clinical collaboration. However, it is important that anaesthetists, in their role as perioperative physicians, are formally included in multidisciplinary team models, particularly with regard to preoperative assessment and optimisation. The reported lack of specialist pre-assessment services in 80% of departments was another finding which requires improvement. This is particularly important as increased access to these services including geriatric input would increase both clinical frailty scoring and cognition screening, two areas where the survey found high variability.

The BGS good practice guideline sets out a number of models for multidisciplinary care in the pre-assessment of older adults. Their guideline distinguishes between geriatrician-led and anaesthetist-led preoperative services (Conroy 2012). We did not make this distinction as we felt that either service would represent adherence to the AAGBI guidelines. It was interesting to note that all but one of the centres that had specialist pre-assessment services were group 3 centres (commonly known as large district general hospitals). A total of 24% (6/25) of our survey respondents were from hospitals that are classified as rural general hospitals; only one of these centres had more than 100 staffed beds (The Scottish Government 2008). It is likely that in these hospitals, given their size and geographical location, that implementation of such services was simply not feasible with the resources available. However, it is unclear why the vast majority of the larger, tertiary care hospitals in Scotland were not providing specialist pre-assessment services. One explanation may be that these hospitals had access to other perioperative resources such as enhanced recovery specialist nurses, and ortho-geriatricians, therefore reducing the need for specialist geriatric pre-assessment.

There are several limitations to the findings of the present survey. We were unable to obtain any information from the largest department in Scotland and one of the largest in the UK.

This represented a significant gap in our dataset. A further limitation is that we began this project during the COVID-19 pandemic. Although it was conducted between ‘waves’, and we were careful to frame all questions as enquiring about ‘normal’ pre-pandemic services, this may well have had an influence. It is likely that centres will have been forced to change practices surrounding their elective work and may have changed service pathways such as those relating to elective and urgent elderly care. This may have contributed to the number of ‘unsure’ responses received. However, an ‘unsure’ response still has some value in that it suggests at the very least a lack of information or communication regarding elderly perioperative care. Therefore, we decided to group these responses with ‘no’, unless the respondent provided free text to suggest otherwise.

## Conclusions

This survey has provided an overview of adherence to perioperative care of the elderly guidelines across Scotland and demonstrated that there are large variations amongst hospitals. Part of this variation could be due to the number of rural general hospitals in Scotland where some of the guideline recommendations might not be practical and achievable in this setting. A UK-wide survey would allow a more comprehensive review of guideline adherence as the dataset would be less influenced by this issue. More specific data on aspects of the published recommendations such as the identification and management of perioperative pain and post-operative delirium in older patients would also be useful.

It would be pragmatic, given the number of rural general hospitals in Scotland, if specific guidelines, or care pathways, applicable to these centres were developed. Telemedicine and video-calling are well-established communication channels, and these services could be supported by the larger district and teaching hospitals. Training and departmental education could also be shared in this way, allowing centralisation of communication and education and facilitating regular service updates.

In conclusion, the present survey has provided a useful starting point for efforts to improve care for older patients presenting for surgery. More emphasis should be placed on forming links between anaesthesia and care of the elderly physicians. The appointment of a lead in geriatric anaesthesia in each department to facilitate these links would be a step in the right direction.

## Supplementary Information

**Additional file 1.**

## Data Availability

The datasets during and/or analysed during the current study available from the corresponding author on reasonable request.

## Abbreviations

NCEPOD: National Confidential Enquiry into Patient Outcome and Death
AAGBI: Association of Anaesthetists of Great Britain and Ireland
COVID-19: Coronavirus disease 2019
CFS: Clinical frailty score
ASA: Association of American Anesthesiologists
BGS: British Geriatric Society
MDT: Multidisciplinary team
ERAS: Enhanced recovery after surgery
