# Supplementary material for: Adherence to published guidelines for perioperative care of the elderly: a survey of Scottish anaesthetic departments
Source: Perioper Med (Lond). 2022 Jul 5;11:26. doi: 10.1186/s13741-022-00258-z (PMC9253247; doi:10.1186/s13741-022-00258-z)
Supplement: Supplementary file 1 — Additional file 1. [file 13741_2022_258_MOESM1_ESM.doc]

1. Do you have a lead clinician for geriatric anaesthesia in your department? Yes / No / Unsure
2. Do you have access to a geriatric specialist who coordinates the perioperative care of the older surgical patient?
   Yes / No / Unsure
3. Do you have a pre-assessment service specifically for elderly or frail patients?
   Yes / No / Unsure
4. Please choose the statement below that best applies: In the last week, what proportion of your department's cases involved patients aged 75 or older?

All or nearly all cases (95% or more)

Most cases (about 75%)

About half of all cases (about 50%)

Some cases (about 25%)

Very few cases (less than 5%)

1. Does your department use a standardised clinical frailty score (e.g. CFS, Edmonton Frail Scale) in the pre-operative assessment of adults over 75?

Yes / No / Unsure

1. Do you specifically screen patients over 75 for cognitive impairment using MOCA / MMSE / 4AT or similar?
   Yes / No / Unsure
2. How often does your department provide the patient or their family with specific information prior to surgery on their risk of developing delirium or neurocognitive decline post-operatively?

Nearly all of the time (>95%)

Frequently (about 75%)

Sometimes (about 50%)

Occasionally (about 25%)

Never or very rarely (less than 5%)

8. How often, prior to Covid-19, were relatives/carers invited to accompany patients with dementia into the theatre department?

Nearly all of the time (>95%)

Frequently (about 75%)

Sometimes (about 50%)

Occasionally (about 25%)

Never or very rarely (less than 5%)

9. Within the last 2 years, has there been any training in your department on caring for elderly / frail and cognitively impaired patients in the peri-operative period?
Yes / No / Unsure
